# Evaluation of ACE-inhibitor Induced Laryngeal Edema Using Fiberoptic Scope: A Case Report

**DOI:** 10.21980/J83P9T

**Published:** 2022-07-15

**Authors:** Joya Singh, Colin Danko

**Affiliations:** * University of Texas Southwestern Medical Center, Department of Emergency Medicine, Dallas, TX

## Abstract

This is a case report of a 55-year-old woman with a past medical history of hypertension who presented to the emergency department with upper lip and cheek swelling isolated to the right side of her face. She was on lisinopril for several years and had never had an episode of swelling in the past. Her vital signs were stable. However, on reassessment, the patient’s right sided swelling progressed to the entire upper lip and she complained of swelling in her submandibular space. Although no swelling was appreciated to the submandibular space on physical exam, and the patient still did not have any signs or symptoms of respiratory distress, the decision was made to use a fiberoptic laryngoscope to evaluate her airway. Fiberoptic laryngoscope showed noticeable laryngeal edema which did not improve after treatment, and the patient was admitted to the medical intensive care unit for close airway observation. This case highlights the use of fiberoptic laryngoscope by emergency physicians in the evaluation of patients with relatively benign appearing angioedema.

**Topics:**

ACE-I, angioedema, fiberoptic laryngoscope, laryngeal edema, tranexamic acid.

**Figure f1-jetem-7-3-v10:**
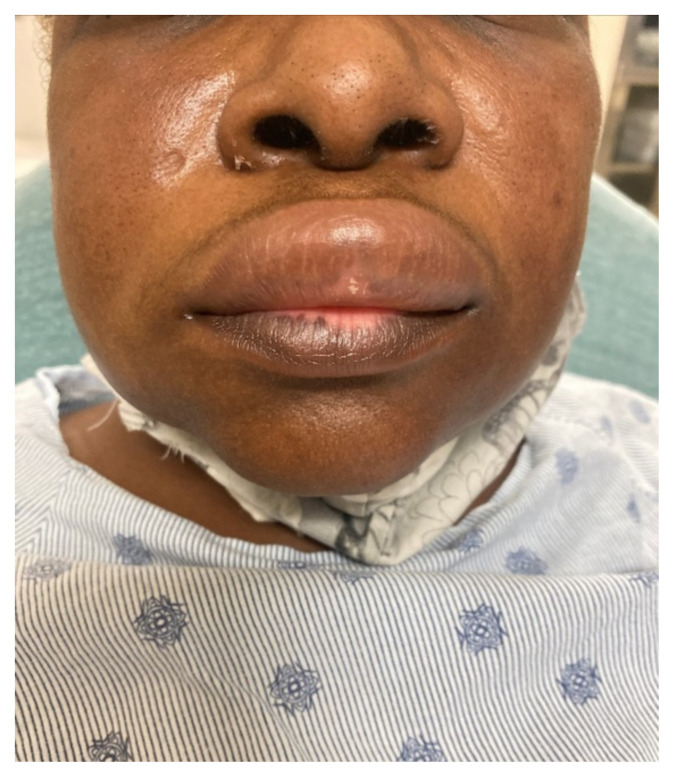


**Figure f2-jetem-7-3-v10:**
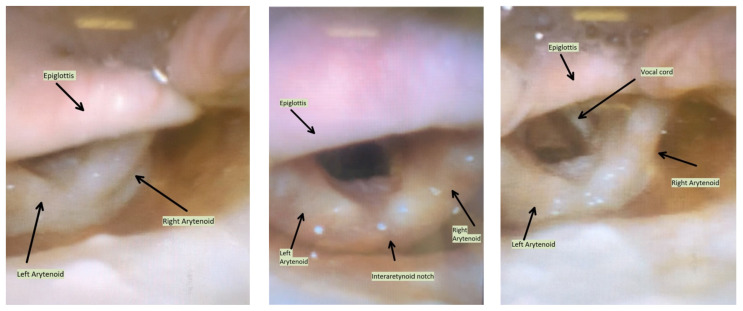
Edema noted along the epiglottis, arytenoids, and vocal cords. Note: epiglottis oriented superiorly/cephalad.

**Figure f3-jetem-7-3-v10:**
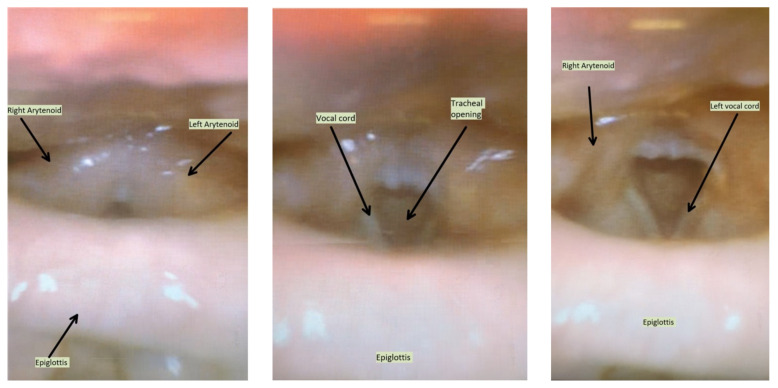
Laryngeal edema noted approximately 4 hours after the initial scope and after 125mg solumedrol, 50mg diphenhydramine by mouth, 50mg diphenhydramine IV, and 2g tranexamic acid. Note: epiglottis oriented inferiorly/caudad.

## Brief introduction

[Fig f1-jetem-7-3-v10][Fig f2-jetem-7-3-v10][Fig f3-jetem-7-3-v10]Angioedema accounts for approximately 110,000 annual visits to the emergency department (ED) each year.^1^ There are different pathophysiological mechanisms that drive angioedema, and the clinical manifestations can vary. The etiology of angioedema includes three general causes: mast-cell mediated allergic angioedema, hereditary angioedema, and angiotensin converting enzyme inhibitor (ACE-I) induced angioedema.^2^ Facial skin is the most likely to be affected by angioedema, but it may also involve the gastrointestinal tract and upper airways, necessitating swift evaluation and intervention by the emergency physician. This case involves a woman who presented to the ED with an isolated swelling of the upper lip and right sided cheek on physical exam, but was found also to have laryngeal edema on evaluation by fiberoptic laryngoscope. Written consent was obtained from the patient for the case report and images.

## Presenting concerns and clinical findings

A 55-year-old African American female with a past medical history of hypertension on lisinopril presented to the ED with upper lip and right sided cheek swelling that began one hour prior to arrival. The swelling had gradually worsened, which prompted her to come for evaluation. She did not have any difficulty breathing, changes in her voice, trouble swallowing, chest tightness, or any gastrointestinal or skin involvement. She noted that she ate a cinnamon roll for breakfast that morning and had experienced some lip swelling after eating cinnamon in the past. She did not have any new changes to her usual diet and routine. She denied any recent changes to her medication.

## Significant findings

Physical exam was initially significant for swelling isolated to the right sided cheek and upper lip. There was no edema to lower lip, uvular swelling, or swelling to the submandibular space. She was speaking full sentences and did not endorse any voice changes. Initial vital signs were as follows: BP 125/77, HR 74, RR 16, and oxygen saturation of 100% on room air. Approximately 40 minutes later, after 125 mg solumedrol intravenous (IV) and 50mg diphenhydramine by mouth, swelling had spread to the entire upper lip and the patient reported spreading to her jaw (Photo 1). Although no jaw or submandibular edema was appreciated on physical exam, a flexible fiberoptic laryngoscope was used to evaluate the patient’s airways given worsening symptoms. Viscous lidocaine was applied intranasally five minutes prior to the procedure. The patient was positioned in a seated position on the stretcher. A flexible fiberoptic laryngoscope was then inserted through the nares and advanced slowly. Laryngoscopy showed diffuse edema of the epiglottis, arytenoids, and ventricular folds (see photos 2–4). Vital signs and respiratory status remained stable both during and after the procedure.

## Patient Course

Given her complaints of progressive symptoms and physical exam findings, she was treated with 1 gram of tranexamic acid. Since facial swelling did not seem to improve two hours after treatment, flexible fiberoptic laryngoscope was used again to evaluate progression of airway involvement (see photos 5–7). There was no improvement noted in the laryngeal angioedema on repeat laryngoscope. Given lack of improvement, she was admitted to the medical intensive care unit (MICU) for close airway monitoring.

She continued to receive cetirizine, IV diphenhydramine, and IV methylprednisolone in the MICU. Her C4 complement was normal (33 mg/dL) and she was believed to have bradykinin-induced angioedema versus mast cell mediated angioedema. After significant improvement in facial swelling the following day, she was discharged home on cetirizine, diphenhydramine, and 40mg prednisone for five days along with instructions to discontinue lisinopril and avoid cinnamon in the future.

## Discussion

Angioedema is a rare condition that causes transient, non-pitting edema due to swelling of the deep dermal, submucosal, and subcutaneous layers of the skin. Although it primarily affects tissues of the face, lips, and tongue, it can also affect extremities, upper respiratory tract, and the gastrointestinal system.^2^ The etiology of angioedema can be explained by three main causes: mast-cell mediated allergic angioedema, hereditary angioedema due to congenital or acquired loss of C1 esterase inhibitor, and angiotensin converting enzyme inhibitor (ACE-I) induced angioedema due to excessive bradykinin.^2^, ^3^ Because the etiology of angioedema presenting to the ED is usually undifferentiated, the general management includes antihistamines and steroids, with the use of intramuscular epinephrine if there are other signs of anaphylaxis.^4^ Angioedema occurs in up to 0.7% of PATIENTS taking ACE-i drugs and is thought to be one of the major causes of nonhereditary angioedema. It also comprises approximately 30% of all angioedema cases presenting to the ED. 5 Given that our patient was on lisinopril, an ACE-i, and reported prior lip swelling with cinnamon consumption, she was treated primarily as ACE-i induced angioedema versus mast-cell mediated angioedema. Steroids and antihistamines were administered to suppress the mast-cell mediated angioedema. She was also treated with tranexamic acid (TXA), an antifibrinolytic agent which inhibits conversion of plasminogen to plasmin. Tranexamic acid is thought to reduce bradykinin mediated angioedema since plasmin is a key step in kallikrein activation and bradykinin formation.^6^

In general, most patients are safe to discharge home after four to six hours of observation if there is no airway involvement, and the localized swelling to face or lips seems to be improving. We can also use the Ishoo staging to help stratify disposition from the ED based on area of angioedema and severity of condition. 7 In general, involvement of the face, lip, and soft palate can mostly be discharged home if swelling is improving. Contrarily, ICU level management is recommended for involvement of the tongue or larynx.^8^ Although our patient was noted to have laryngeal angioedema, she did not report any dyspnea or throat swelling, and was maintaining her oxygen saturation well over 96% without any signs of respiratory distress. Therefore, emergently securing an airway at that time was not indicated. However, she was admitted to the MICU for close airway observation. This case highlights the role of early evaluation of airway involvement using fiberoptic laryngoscope to identify airway involvement of angioedema. Further monitoring in the ED with additional medications and monitoring in the ICU yielded a favorable outcome for our patient.

## Conclusion

This is a case of angioedema of the upper lip that also had laryngeal involvement that was noted only upon evaluation by a flexible fiberoptic laryngoscope. It is important for the emergency physician to anticipate potential airway compromise, and early use of a flexible fiberoptic laryngoscope can help us identify progression of laryngeal angioedema. In addition, this case also provides visual guidance for junior residents to help identify laryngeal involvement of angioedema.

## Supplementary Information


























