# Supplementary figures and images for: Evaluation of ACE-inhibitor Induced Laryngeal Edema Using Fiberoptic Scope: A Case Report
Source: J Educ Teach Emerg Med. 2022 Jul 15;7(3):V10–3. doi: 10.21980/J83P9T (PMC10332706; doi:10.21980/J83P9T)

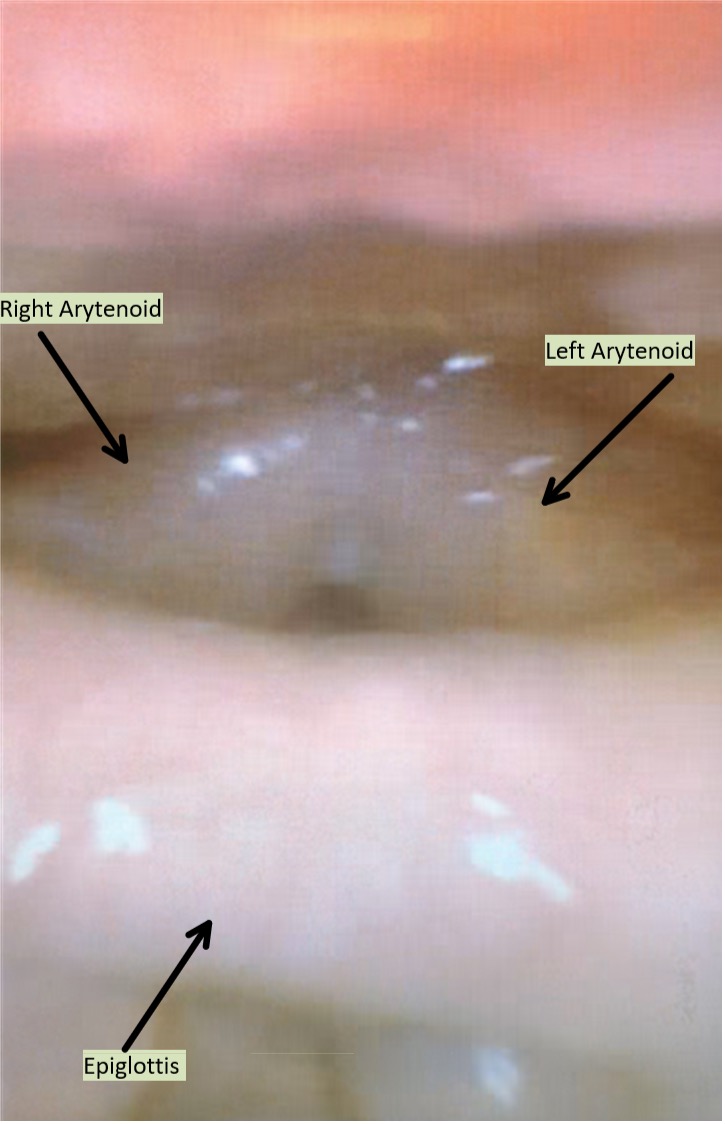

Supplement: Supplementary file 1 [file jetem-7-3-v10-supp1.jpg]

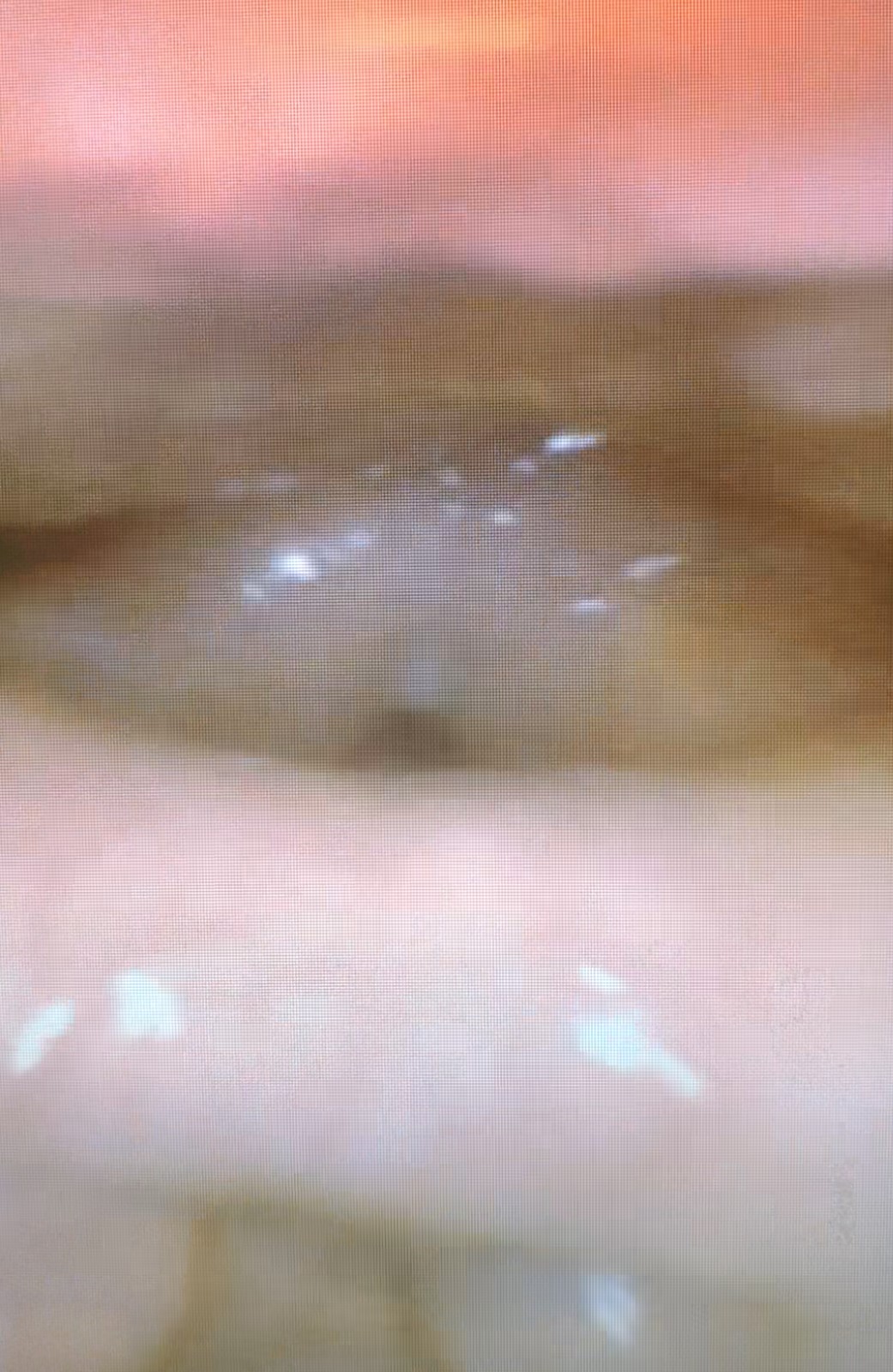

Supplement: Supplementary file 2 [file jetem-7-3-v10-supp2.jpg]

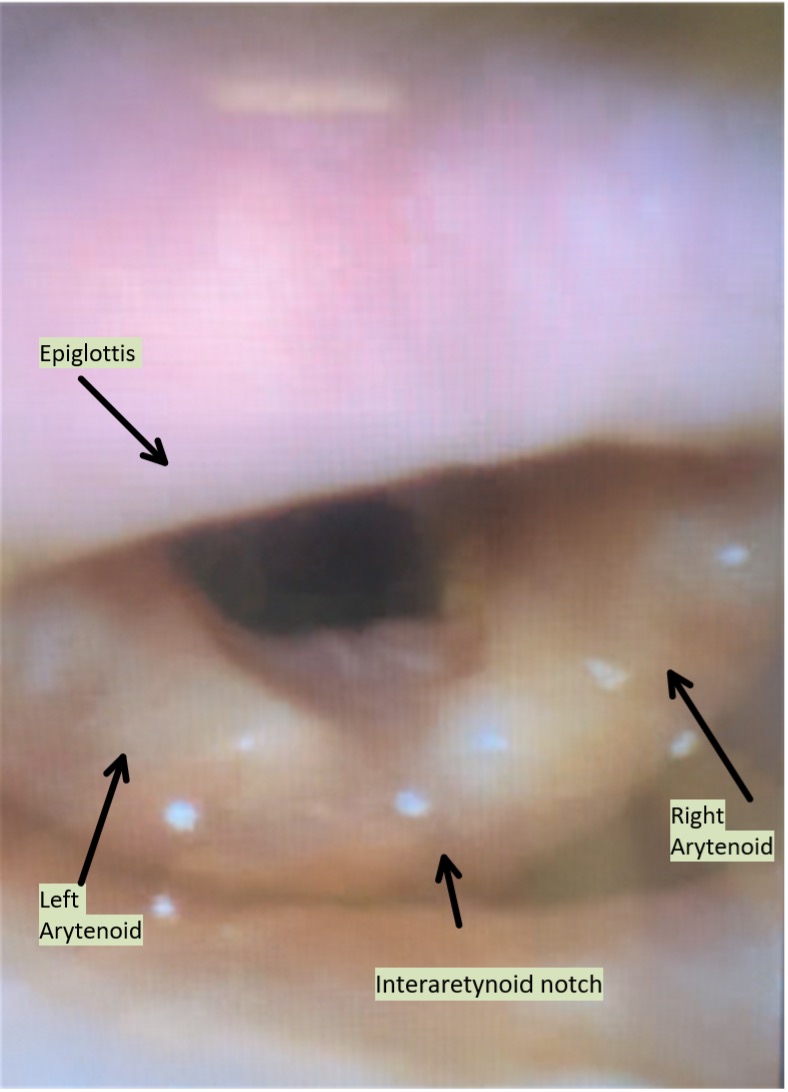

Supplement: Supplementary file 3 [file jetem-7-3-v10-supp3.jpg]

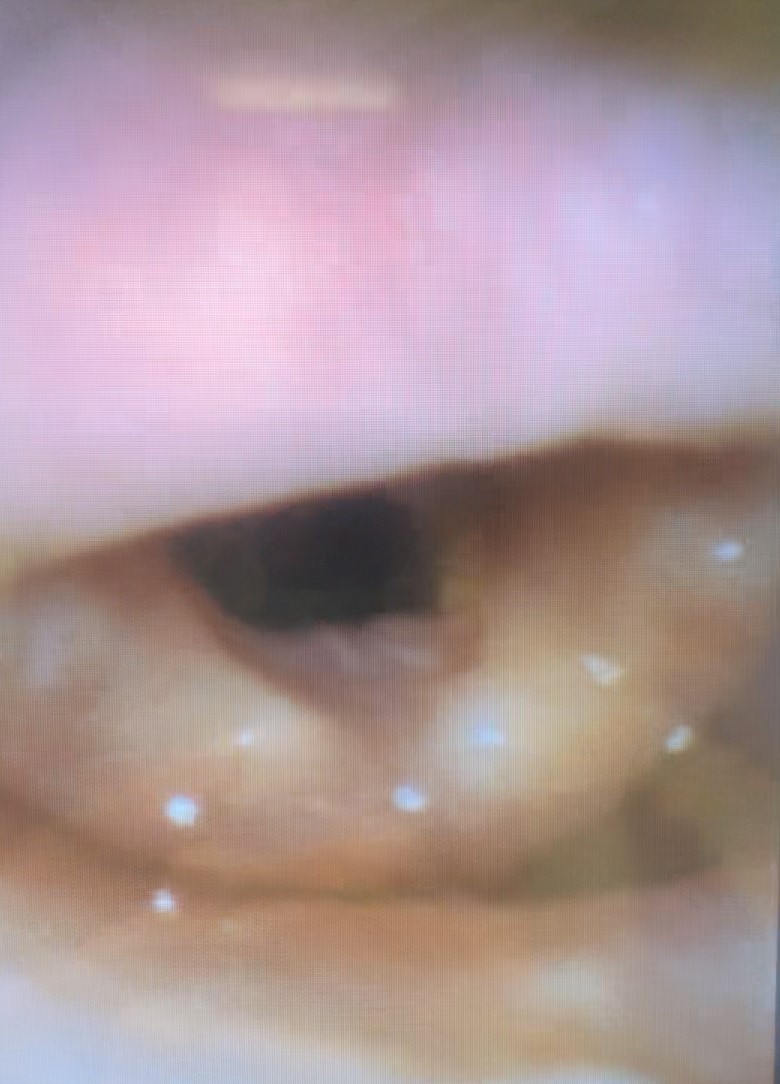

Supplement: Supplementary file 4 [file jetem-7-3-v10-supp4.jpg]

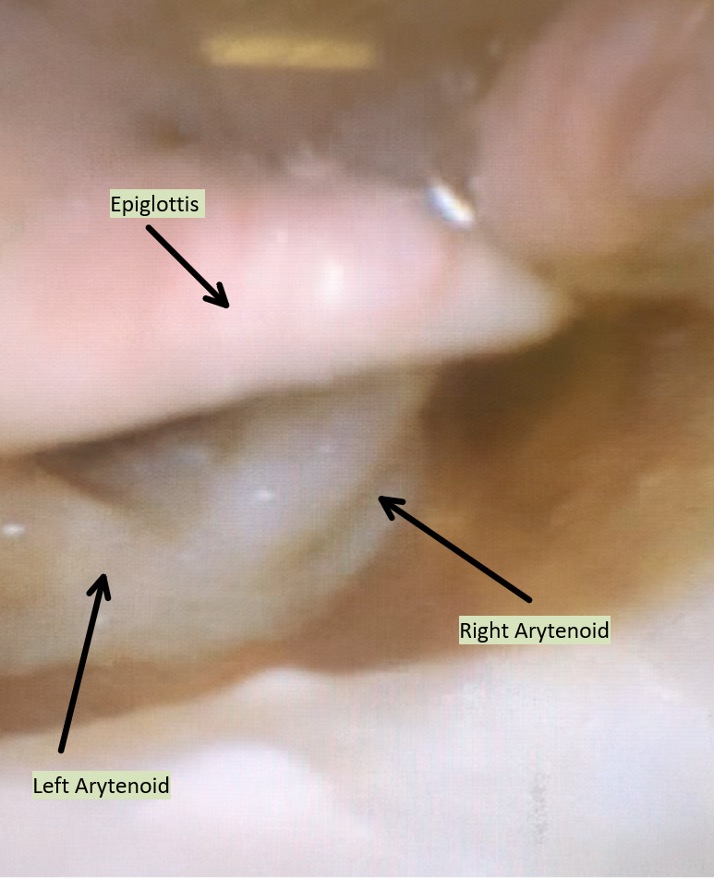

Supplement: Supplementary file 5 [file jetem-7-3-v10-supp5.jpg]

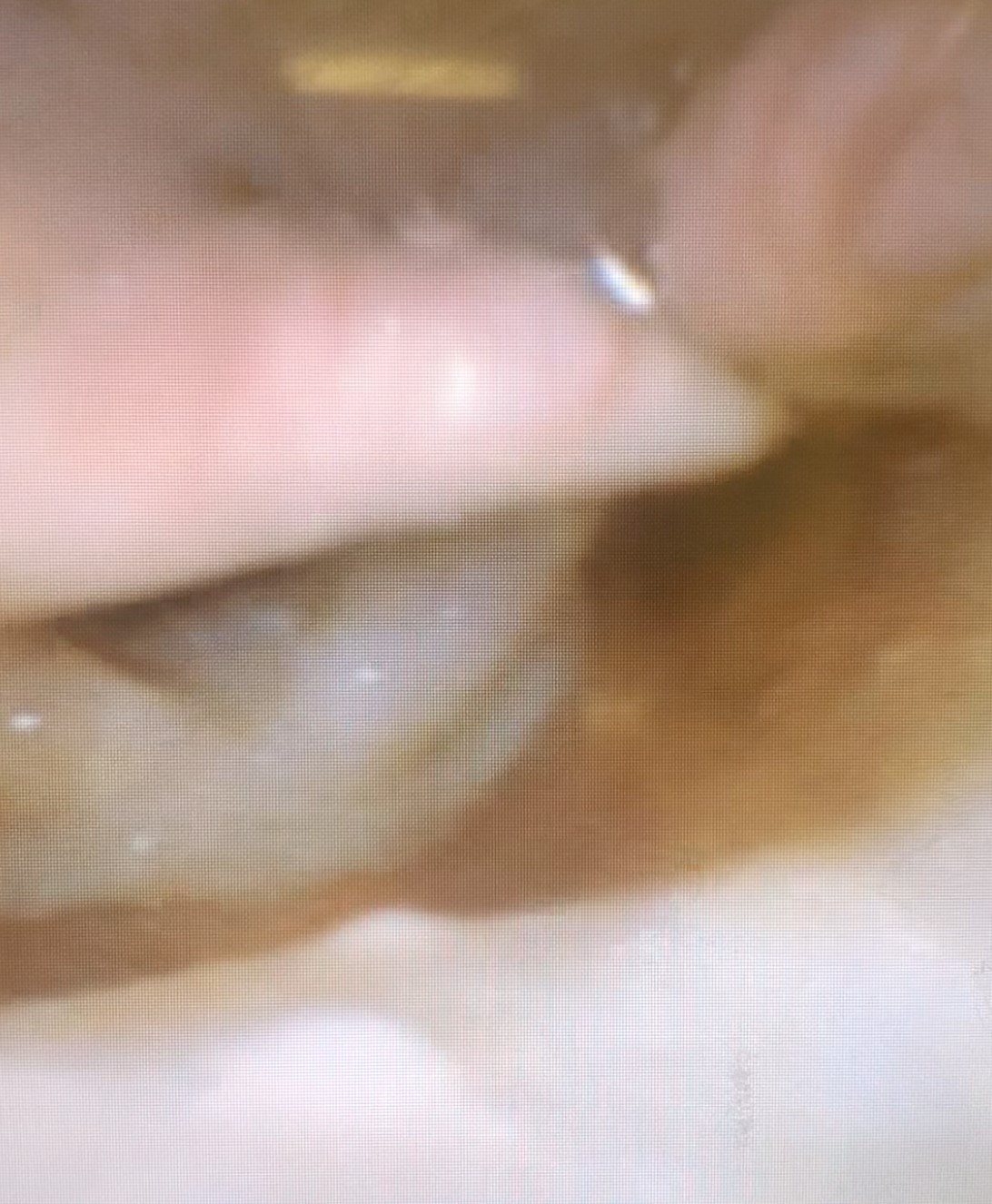

Supplement: Supplementary file 6 [file jetem-7-3-v10-supp6.jpg]

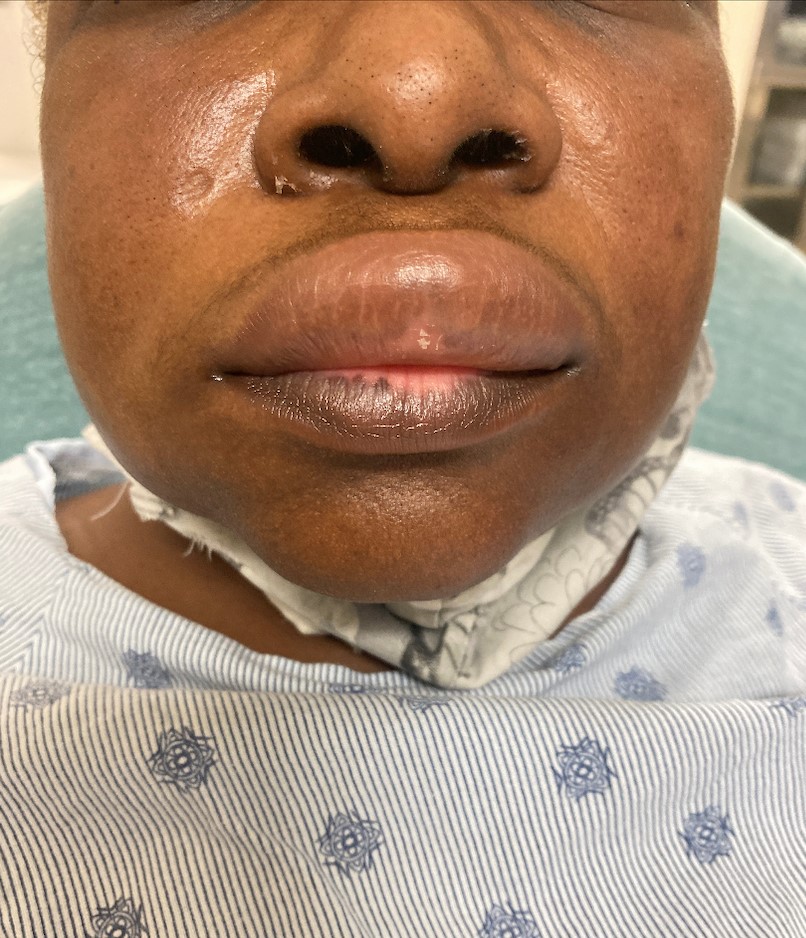

Supplement: Supplementary file 7 [file jetem-7-3-v10-supp7.jpg]

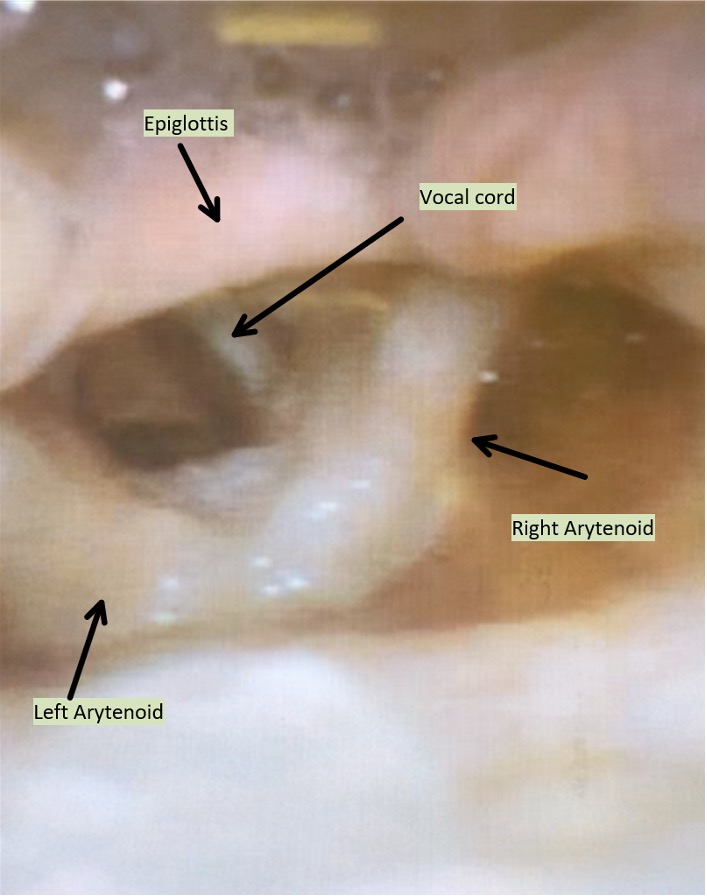

Supplement: Supplementary file 8 [file jetem-7-3-v10-supp8.jpg]

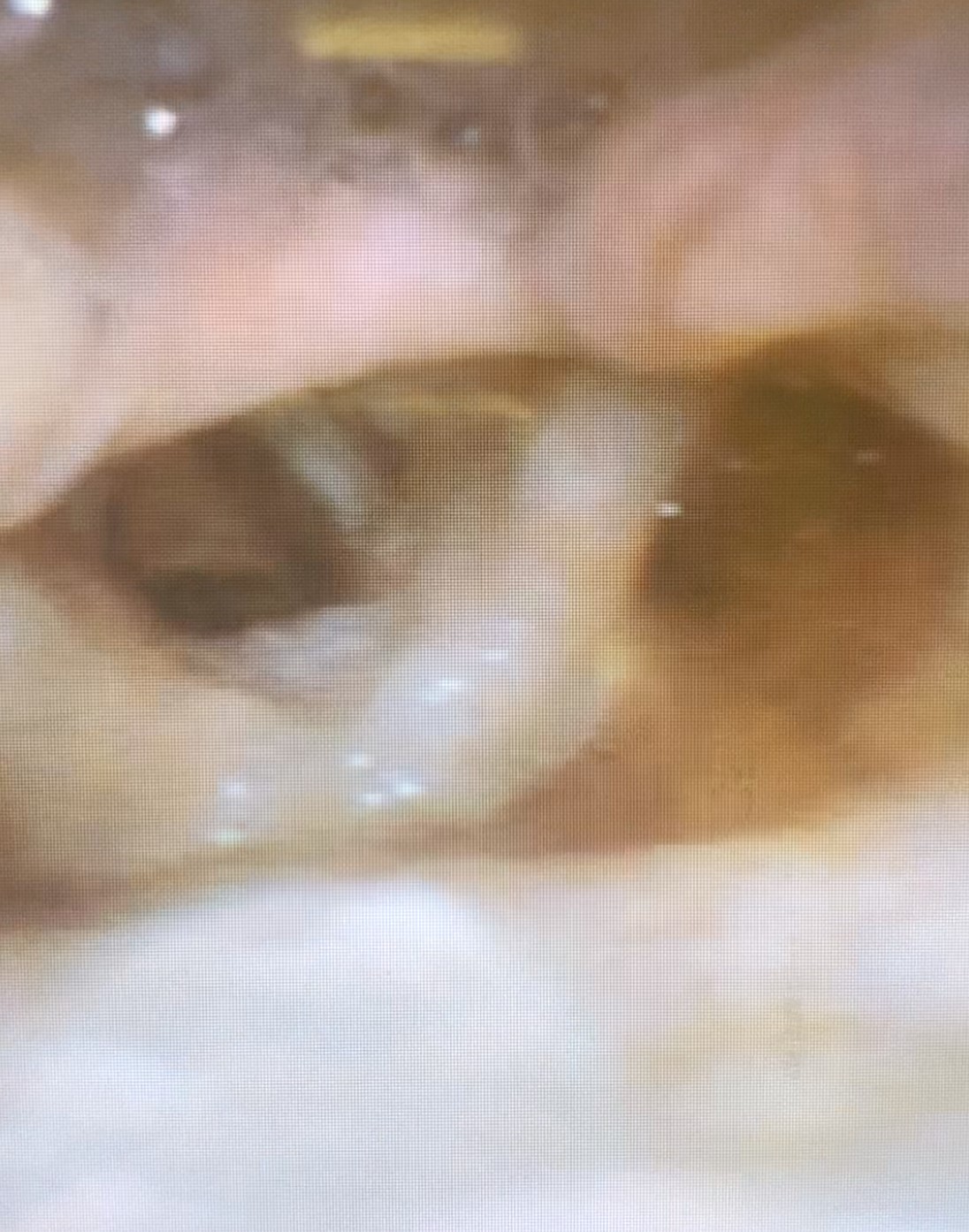

Supplement: Supplementary file 9 [file jetem-7-3-v10-supp9.jpg]

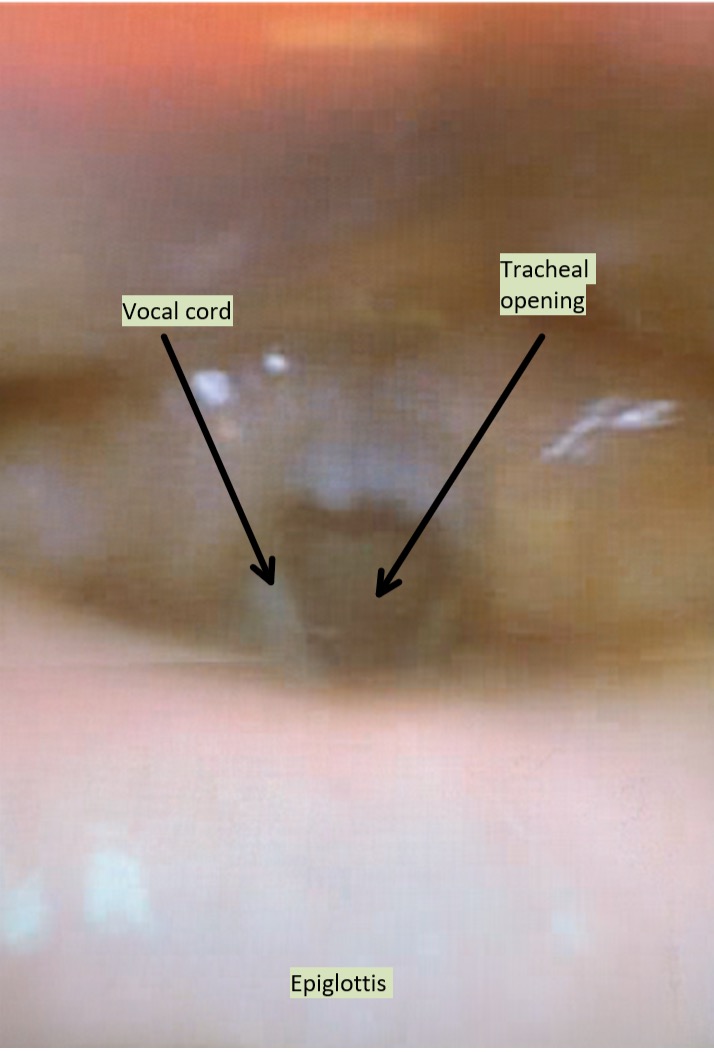

Supplement: Supplementary file 10 [file jetem-7-3-v10-supp10.jpg]

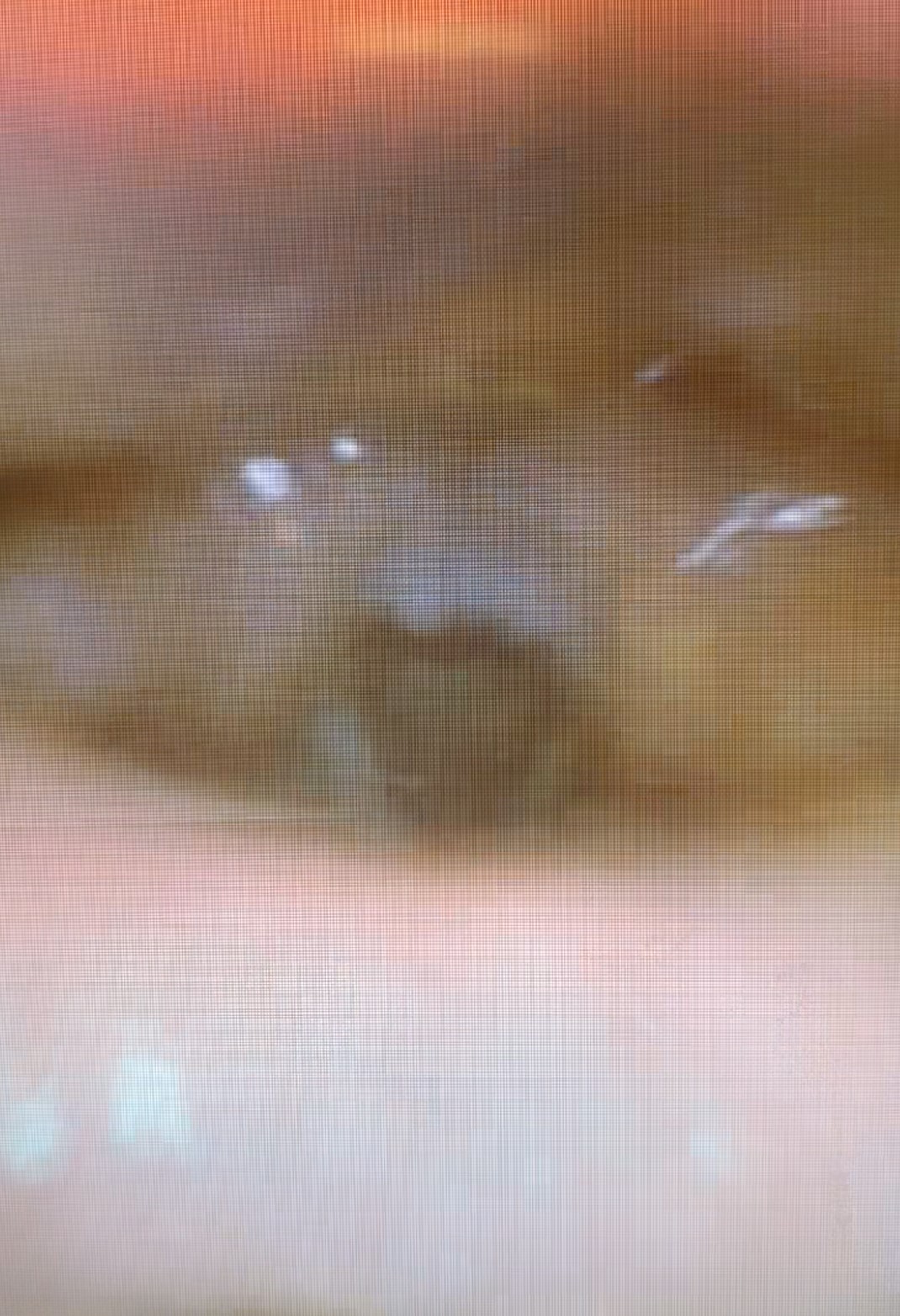

Supplement: Supplementary file 11 [file jetem-7-3-v10-supp11.jpg]

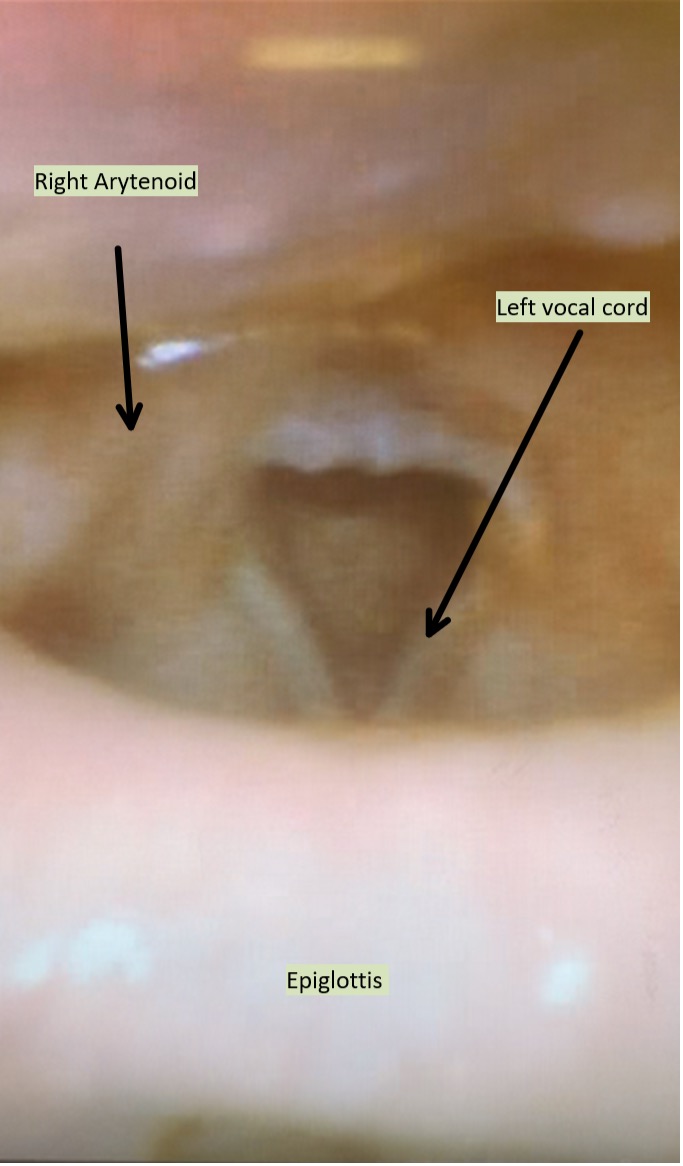

Supplement: Supplementary file 12 [file jetem-7-3-v10-supp12.jpg]

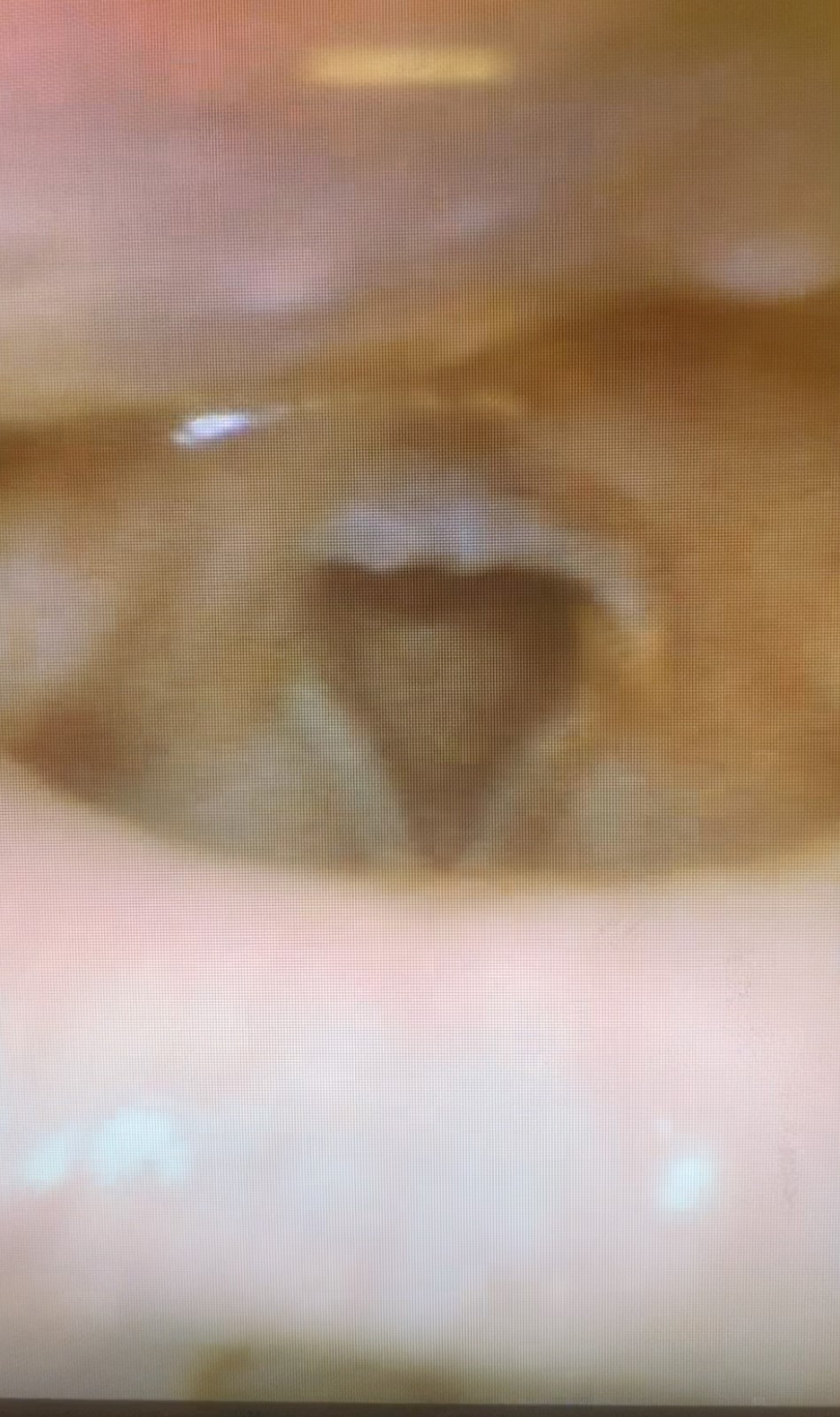

Supplement: Supplementary file 13 [file jetem-7-3-v10-supp13.jpg]
